# Primary Rosai-Dorfman Disease of Bone: A Report of Two Cases

**DOI:** 10.1155/2019/1720131

**Published:** 2019-01-03

**Authors:** Andrew B. Ross, Kirkland W. Davis, Darya Buehler, Brian Y. Chan

**Affiliations:** ^1^Department of Radiology, University of Wisconsin School of Medicine and Public Health, 600 Highland Ave. MC 3252, Clinical Science Center, Madison, WI 53792, USA; ^2^Department of Radiology, University of Wisconsin School of Medicine and Public Health, Madison, WI, USA; ^3^Department of Pathology and Laboratory Medicine, University of Wisconsin School of Medicine and Public Health, Madison, WI, USA; ^4^Department of Radiology, University of Utah, Salt Lake City, UT, USA

## Abstract

Rosai-Dorfman disease (RDD), sometimes known as sinus histiocytosis with massive lymphadenopathy, is a rare histiocytic disorder that most commonly presents as painless, massive cervical lymphadenopathy in young adults. Extranodal disease can occur in up to 40% of patients but primary involvement of bone is rare. We present two cases of primary RDD of bone: one case of multifocal osseous RDD presenting as a painful lesion in the elbow, and one case of a solitary osseous lesion presenting as a painful lesion in the wrist.

## 1. Introduction

Originally described in 1969 [1], RDD traditionally has been classified as one of the non-Langerhans cell histiocytoses, a group of rare disorders characterized by the overproduction and accumulation of cells from macrophage-dendritic lineage. A more recent revision of the histiocytosis classification has proposed that the characteristics of RDD are sufficiently unique for it to merit its own category within the histiocytic disorders [2]. The most common clinical presentation of the disorder is of massive, painless cervical lymphadenopathy in children and young adults [3]. Accompanying constitutional symptoms may be present with the clinical picture mimicking lymphoma. Extranodal involvement is common, occurring in up to 40% of patients, and is particularly prevalent in older patients with the disease [4]. The most common sites of extranodal involvement include the skin, orbits, central nervous system, upper respiratory tract, and occasionally the gastrointestinal tract [5]. Bone involvement as a secondary site of RDD is thought to occur in less than 10% of patients [4]. Osseous involvement without lymphadenopathy (primary disease of bone) is rarer still and has been reported in only a small number of patients [6–9]. Primary osseous disease presents as a solitary lesion in the majority of patients [10]. We present two cases of primary osseous RDD. Case 1 is a rare case of a 76-year-old woman with multifocal primary RDD of bone presenting with a painful elbow lesion. Case 2 is a 20-year-old man presenting with a solitary painful osseous lesion in the wrist.

## 2. Case Presentations

### 2.1. Case 1

**Figure 1:**
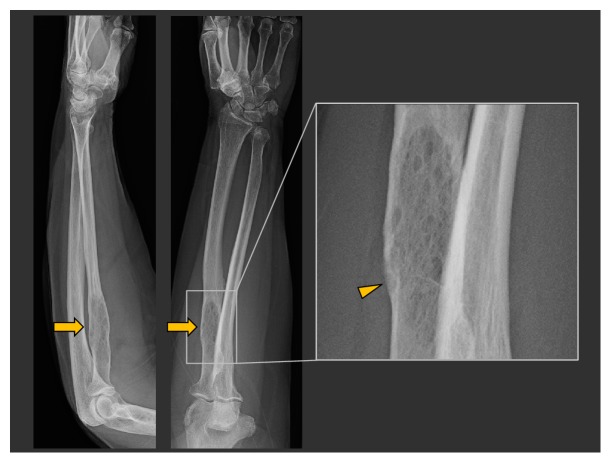
Lateral and AP views of the left forearm from patient 1 show a mildly expansile lucent lesion in the proximal left radial diaphysis (arrows). The zoomed in view better shows the cortical thinning and internal septations but no periosteal reaction or obvious soft tissue mass.

The patient is a 76-year-old woman with a two-year history of left elbow pain empirically diagnosed as gout. When her symptoms failed to improve with appropriate management, radiographs were obtained, demonstrating a lesion in the proximal radius (Figure 1) characterized as a mildly expansile lucent lesion with a thin zone of transition but no sclerotic rim. Internal osseous septations were present and there was cortical thinning but no visible cortical breakthrough, periosteal reaction, calcified matrix, or soft tissue mass. The initial differential diagnosis included metastasis, multiple myeloma, and other less common entities such as a primary sarcoma of bone or atypical infectious process. She was referred to our tertiary care hospital to consult with an oncologic orthopedic surgeon. Further history obtained at that clinic visit elicited that 3 years previously she had incidentally discovered lytic lesions in her skull and left clavicle that were evaluated in another medical system. Biopsy of both lesions performed at that time was inconclusive showing a mix of inflammatory and fibrous cells per report. The pathologic specimens were not available for further review. Physical exam at her clinical visit was unremarkable with no palpable lymphadenopathy and no visible abnormality at the symptomatic left elbow. SPEP and UPEP tests were negative.

**Figure 2:**
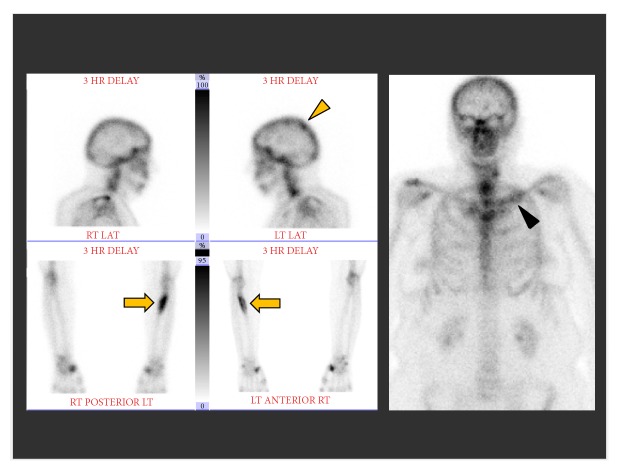
Nuclear medicine bone scan shows marked radiotracer uptake within the lesion in the proximal left radius (orange arrows), within the calvarium (orange arrowhead), and medial left clavicle (black arrowhead).

**Figure 3:**
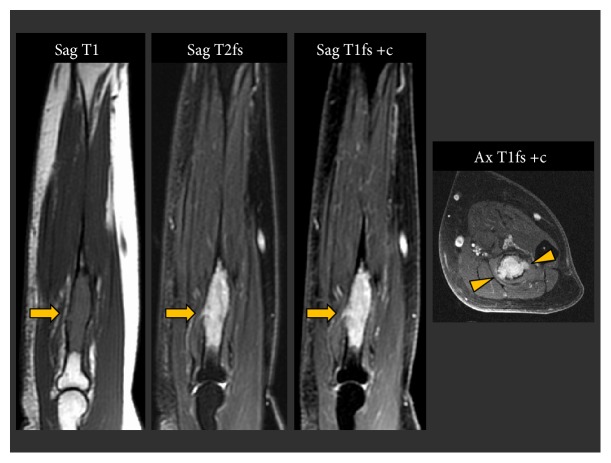
Sagittal T1, T2 fat-suppressed, and sagittal and axial T1 fat-suppressed postcontrast images of the left forearm demonstrate a T1 isointense, T2 hyperintense, avidly enhancing lesion (arrows). The axial postcontrast image demonstrates the extent of the cortical thinning with focal areas of cortical breakthrough. There is no surrounding edema or abnormal soft tissue enhancement.

Her initial imaging work-up included CT of the chest, abdomen, and pelvis; contrast-enhanced MRI of the left forearm; and nuclear medicine bone scan. Her CT scan showed no findings of primary malignancy and—pertinent to her eventual diagnosis—showed no lymphadenopathy or vital organ abnormality. Bone scan demonstrated marked radiotracer uptake at the site of the lytic lesion in the proximal left radius as well as at the previously biopsied skull and left clavicle lesions (Figure 2). The MR scan of the left forearm showed a marrow replacing lesion within the proximal diaphysis of the radius (Figure 3). The lesion was T1 isointense, T2 hyperintense and demonstrated avid enhancement. Cortical thinning and small areas of cortical breakthrough not visible on the radiographs were apparent on the MRI. No associated soft tissue mass or perilesional edema was present.

**Figure 4:**
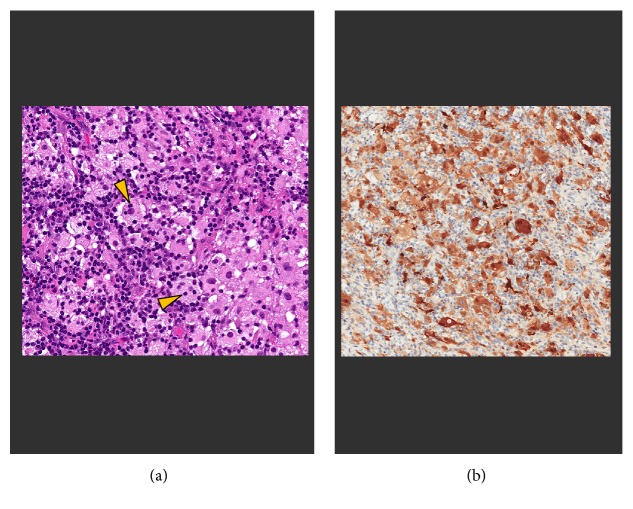
Histology from open biopsy of the proximal radius lesion demonstrates sheets of pale histiocytic cells in an inflammatory background (a). The histiocytes demonstrate emperipolesis (engulfment of intact lymphocytes within the cytoplasm—arrowheads). The histiocytes express S100 protein by immunohistochemistry, characteristic of Rosai-Dorfman disease (b).

**Figure 5:**
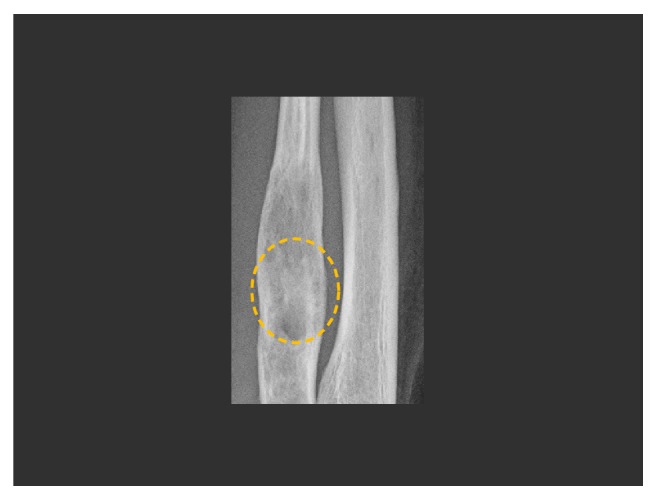
A follow-up radiograph 8 weeks after surgery demonstrates partial filling in of the lucent lesion with healing bone (dashed circle) following curettage.

At the request of the orthopedic oncologist, a fluoroscopy-guided percutaneous biopsy was performed by Musculoskeletal Interventional Radiology. This rendered only tiny fragments of tissue that were nondiagnostic at histologic review. The patient then underwent open biopsy and curettage of the lesion with Orthopedic Surgery for both diagnostic and treatment purposes. Lesion histology demonstrated features diagnostic of RDD including emperipolesis (engulfment of intact lymphocytes contained with the cytoplasm of histiocyte cells) and positive S100 immunohistochemical staining (Figure 4). At her follow-up clinic visit 8 weeks after surgery, the patient reported resolution of her left elbow pain, and repeat radiographs demonstrated partial filling in of the lesion with healing bone (Figure 5). She was discharged from clinic and instructed to follow up if she developed recurrent left elbow symptoms or similar symptoms at a new site. One year later, she has not sought further care at our institution.

### 2.2. Case 2

**Figure 6:**
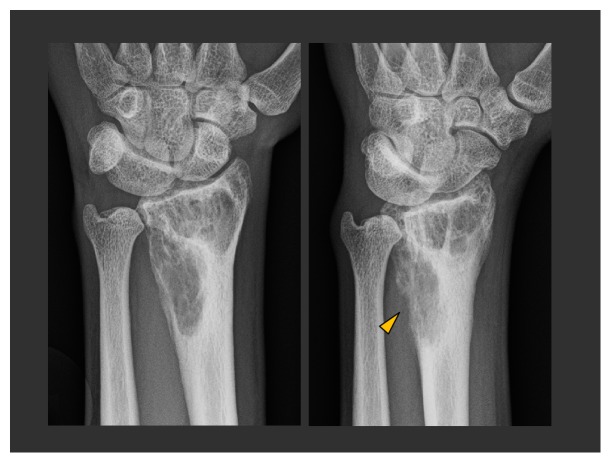
PA and oblique views of the left wrist from patient 2 demonstrate a lucent lesion with numerous internal septations in the distal radial metaphysis and epiphysis. There is marked thinning of the cortex along the radial margin of the distal radius (arrowhead). The cortical thinning is more pronounced and the lesion more aggressive in appearance than in patient 1.

**Figure 7:**
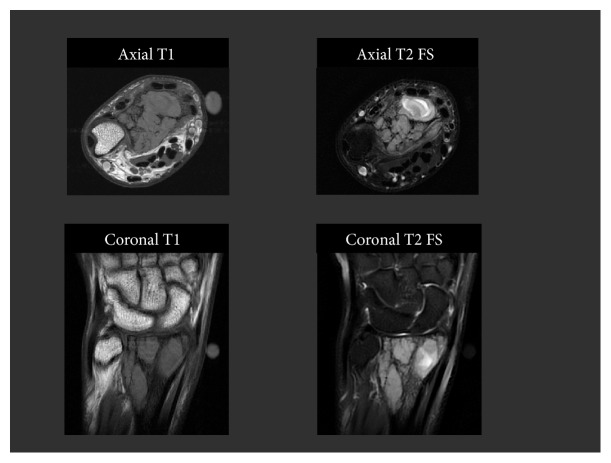
Axial and coronal T1 and T2 fat-suppressed images through the left wrist demonstrate the lesion to be isointense on T1 and heterogeneously hyperintense on T2 with numerous internal septations.

The patient is an otherwise healthy 20-year-old incarcerated man who presented with a 1-year history of intermittent left wrist pain. Left wrist radiographs obtained as part of his initial evaluation (Figure 6) demonstrated a mildly expansile mixed lucent and sclerotic lesion in the distal left radius with multiple internal septations. The zone of transition was less well defined than in case 1 and a greater degree of cortical thinning was evident. He was referred to our facility for further evaluation. Similar to patient 1, physical exam at his clinical visit was unremarkable with no abnormality of the left wrist and no palpable lymphadenopathy. The clinical history did not reveal any additional symptoms beyond his intermittent left wrist pain. Review of a noncontrast MRI of the left wrist from an outside institution (Figure 7) demonstrated a multilobular, septated marrow replacing lesion in the distal radial metaphysis and epiphysis with more heterogenous signal characteristics than seen in case 1. Again, no soft tissue mass or perilesional edema was present

The patient was taken directly to open biopsy, curettage, and bone grafting with Orthopedic Surgery. Intraoperative frozen sections demonstrated an inflammatory proliferation with final diagnosis deferred to permanents. Final histologic analysis showed the same characteristic features of osseous RDD described in case 1. One month of follow-up after surgery, the patient has had relief from his wrist pain and will be followed up expectantly.

## 3. Discussion

RDD is a rare, nonmalignant histiocytic disorder with an as yet unknown etiology. Infectious causes—including human herpes virus, parvovirus B19, and Epstein-Barr virus—have been proposed but with conflicting experimental results [11–14]. An association with IgG4 disease has also been suggested but proven inconsistent [2]. Recurrent* KRAS* and* MAP2K1* mutations have been found in approximately one-third of sporadic RDD cases suggesting that at least a subset of these lesions represent clonal proliferations [15]. Based on the various clinicopathologic associations, RDD is now classified into sporadic classical (nodal), extranodal, familial, neoplasia-associated, and immune disorder-associated forms. The classical clinical presentation is massive painless cervical lymphadenopathy in a young adult frequently with associated constitutional symptoms, but extranodal involvement, often as a subcutaneous mass, is common at 40% [3, 4]. Bone involvement has been estimated at 10%, usually as a secondary process in patients with other sites of disease [4]. Primary involvement of bone is rare, estimated at 2-8% of cases [16]. Primary osseous RDD typically is solitary and has been reported in the femur, tibia, skull, clavicle, sacrum, and small bones of the hands and feet [8, 10, 16].

Patient 1 presented with a polyostotic process with a history of lesions in the skull and clavicle. Although the previous histology of the skull and clavicle lesions was inconclusive, the reported proliferation of inflammatory and fibrous cells is consistent with RDD and may represent sampling error, a known diagnostic pitfall of this entity. Further evaluation with immunohistochemical staining may have been helpful as the expression of S-100 is characteristic of RDD [17]. Emperipolesis is also characteristic but variably present [18]. In this patient, the previously biopsied lesions had similar imaging characteristics to the diagnostic lesion in her radius and her work-up for multiple myeloma or a primary malignancy was negative. This suggests that the lesions in the calvarium and clavicle may also have represented RDD. At the age of 76, she is much older than patients in previous cases series of RDD of bone that have reported a mean age of 31 for patients with osseous involvement [6]. Patient 2, at 20 years of age and with a solitary lesion, had a pattern of disease more in keeping with the previous literature on primary RDD of bone [10].

The imaging manifestations of RDD in bone are not specific to the disease. On radiographs, osseous lesions typically present with a lytic appearance with variably defined margins ranging from sclerotic to permeative [10]. Periostitis, as seen in case 2, is rare. Cortical thinning and focal breakthrough, as seen in both cases, are common. Purely sclerotic lesions have been reported but are thought to be exceedingly unusual [19]. The appearance—both on radiographs and MRI—may overlap with multiple myeloma or metastasis in the older patient and Langerhans cell histiocytosis or osteomyelitis in the younger patient, among other differential diagnostic possibilities. The majority of reported lesions have been painful but this may reflect a bias towards the detection of symptomatic sites of disease [6]. A dense inflammatory infiltrate may be more prevalent within the lesion than the characteristic histiocytes leading to sampling error and complicating histologic diagnosis from percutaneous biopsy specimens [5]. Indeed, our first patient had three nondiagnostic percutaneous biopsies prior to being diagnosed correctly after open biopsy. If RDD is contemplated in the differential, care must be taken to obtain adequate tissue for diagnosis during any percutaneous biopsy attempt.

There is no consensus treatment algorithm for primary RDD of bone. In general, RDD frequently requires no treatment and may spontaneously regress in as many as 80% of cases [20]. However, mortality has been reported in cases of vital organ involvement and secondary involvement of bone has been reported as a marker of increased risk of death [4]. In such cases with systemic involvement, treatment options include surgical resection when possible, corticosteroids, rituximab, and a variety of chemotherapeutic agents [5, 21]. Primary RDD of bone is not thought to pose a mortality risk and treatment is focused on palliating sites of painful disease or preventing complications such as pathologic fracture. Surgical resection or curettage and bone grafting are the most commonly described interventions in these instances [10]. There is no agreed upon process for disease surveillance and many patients are managed expectantly with further imaging obtained if symptoms recur or new symptoms develop.

Primary RDD of bone is an unusual manifestation of a rare disease. The imaging appearance is nonspecific and sampling error may complicate diagnosis on the basis of percutaneous biopsy leading to delays in diagnosis and patient care. Knowledge of this uncommon entity may assist the radiologist in making a timely diagnosis.
